# Selective estrogen receptor modulator lasofoxifene suppresses spondyloarthritis manifestation and affects characteristics of gut microbiota in zymosan-induced SKG mice

**DOI:** 10.1038/s41598-021-91320-1

**Published:** 2021-06-07

**Authors:** Hyemin Jeong, In Young Kim, Eun-Kyung Bae, Chan Hong Jeon, Kwang-Sung Ahn, Hoon-Suk Cha

**Affiliations:** 1 Department of Internal Medicine, Soonchunhyang University Hospital, Bucheon, Republic of Korea; 2 Department of Internal Medicine, National Police Hospital, Seoul, Republic of Korea; 3 Samsung Biomedical Research Institute, Seoul, Republic of Korea; 4 Functional Genome Institute, PDXen Biosystems Inc, Seoul, Republic of Korea; 5 Department of Medicine, Samsung Medical Center, Sungkyunkwan University School of Medicine, 81 Irwon-Ro Gangnam-gu, Seoul, 06351 Republic of Korea

**Keywords:** Rheumatology, Spondyloarthritis

## Abstract

Ankylosing spondylitis is a male-predominant disease and previous study revealed that estrogens have an anti-inflammatory effect on the spondyloarthritis (SpA) manifestations in zymosan-induced SKG mice. This study aimed to evaluate the effect of selective estrogen receptor modulator (SERM) lasofoxifene (Laso) on disease activity of SpA. Mice were randomized into zymosan-treated, zymosan + 17β-estradiol (E2)-treated, and zymosan + Laso-treated groups. Arthritis was assessed by ^18^F-fluorodeoxyglucose (^18^F-FDG) small-animal positron emission tomography/computed tomography and bone mineral density (BMD) was measured. Fecal samples were collected and 16S ribosomal RNA gene sequencing was used to determine gut microbiota differences. Both zymosan + E2-treated mice and zymosan + Laso-treated mice showed lower arthritis clinical scores and lower ^18^F-FDG uptake than zymosan-treated mice. BMD was significantly higher in zymosan + E2-treated mice and zymosan + Laso-treated mice than zymosan-treated mice, respectively. Fecal calprotectin levels were significantly elevated at 8 weeks after zymosan injection in zymosan-treated mice, but it was not significantly changed in zymosan + E2-treated mice and zymosan + Laso-treated mice. Gut microbiota diversity of zymosan-treated mice was significantly different from zymosan + E2-treated mice and zymosan + Laso-treated mice, respectively. There was no significant difference in gut microbiota diversity between zymosan + E2-treated mice and zymosan + Laso -treated mice. Laso inhibited joint inflammation and enhanced BMD in SKG mice, a model of SpA. Laso also affected the composition and biodiversity of gut microbiota. This study provides new knowledge regarding that selected SpA patients could benefit from SERM treatment.

## Introduction

Ankylosing spondylitis (AS) is a male-predominant disease and male gender is associated with more severe radiographic progression^1,2^. Male patients progressed from non-radiographic axial spondyloarthritis (SpA) to AS more frequently than female patients^3^. The underlying pathogenesis of the effect of sex on the AS is still uncertain. Sex hormones interact with genetic and environmental factors and determine expression of cell markers involved in innate and adaptive immunity^4^. Females produce higher Th2 responses, whereas male generate more Th17 response. Jimenez-Balderas et al.^5^ reported that in menstruating patients, estradiol levels were lower significantly in patients with active AS than those of patients with inactive AS. However, there was no significant association between estrogens and spinal radiographic progression in patients with AS^6^. In a human study, estrogen levels are affected by several factors including age, sex, and bone marrow density. We hypothesized that female sex hormone might affect on SpA activity, and we found that estrogens attenuate SpA manifestations using SKG mice^7^. However, estrogens treatment causes various side effects, including increased risk of breast cancer, endometrial cancer, and thromboembolism. Selective estrogen receptor modulators (SERMs) were used to treat osteoporosis without side effects of estrogens. Experiment to evaluate the effect of SERMs in the SpA is needed.

The SKG mouse is a BALB/c strain with a W163C mutation of ZAP-70, which develops spontaneous autoimmune inflammatory arthritis after β-glucan exposure^8^. Although the SKG mouse was first used as rheumatoid arthritis mouse model, Ruutu et al. reported that SKG mice have clinical features of SpA and Crohn`s disease like-ileitis^9^. And we found that zymosan injected SKG mice developed SpA features including axial joint inflammation, peripheral arthritis, and psoriatic skin lesion^10^. Intestinal inflammation was also found in zymosan injected SKG mice. It is well known that gut inflammation is involved in the pathogenesis of AS. Circulating gut-derived interleukin (IL)-23 may be responsible for joint inflammation^11^. Altered microbiota composition is implicated in the pathogenesis of inflammatory SpA. Fecal microbiota in patients with SpA revealed specific dysbiosis was found compared to healthy control^12^. Gut microbial alterations were related to the development of autoimmune uveitis^13^. The purpose of this study was to evaluate the effect of SERM lasofoxifene (Laso) on disease activity of SpA using an animal model. To further interrogate association between microbiota and SpA, we performed fecal microbiota metagenomics analysis.

## Results

### Laso treatment ameliorate arthritis in SKG mice

**Figure 1:**
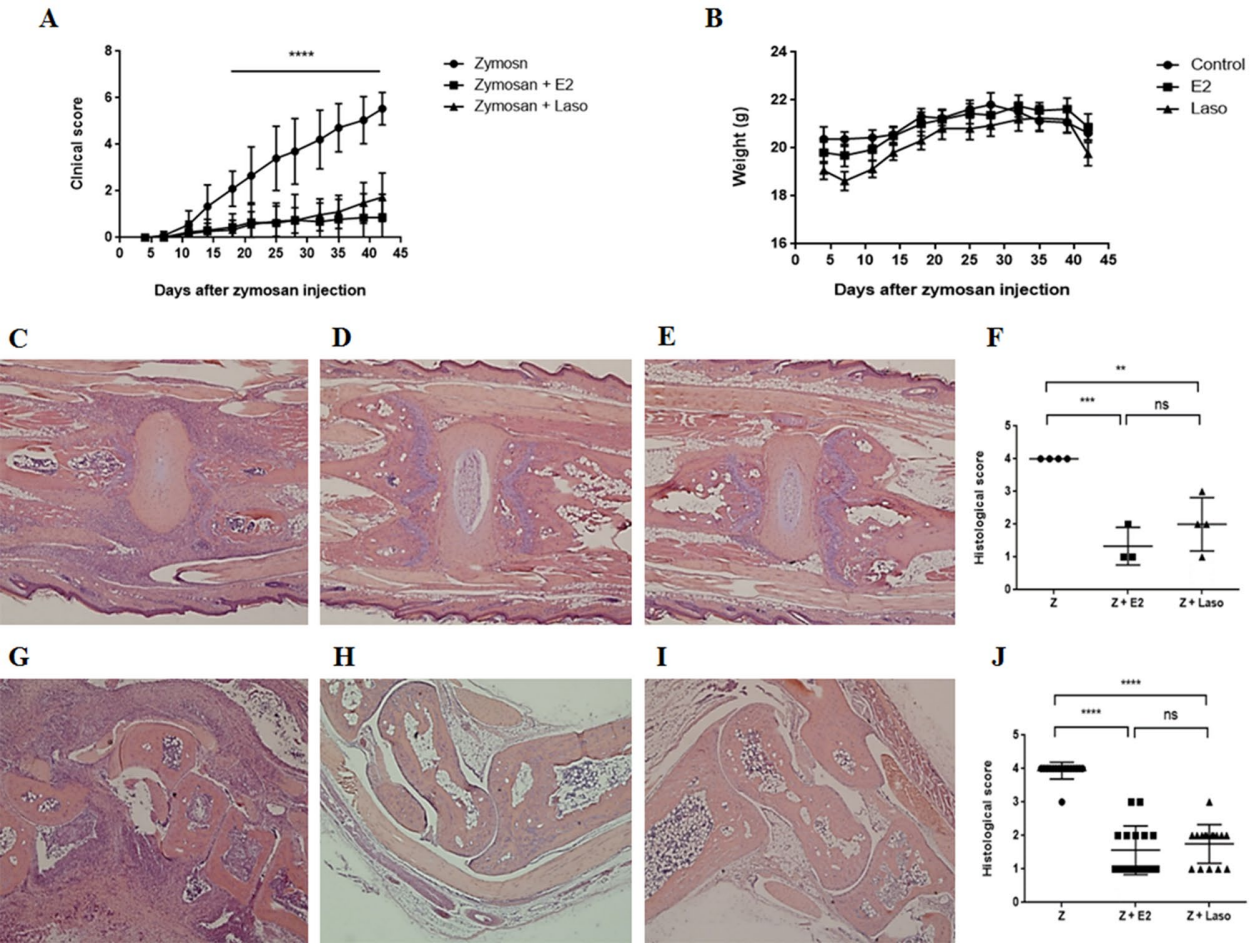
Lasofoxifene (Laso) treatment suppressed the development of arthritis in SKG mice. (**A**) Clinical scores were lower in zymosan (Z) + 17β-estradiol (E2) or Z + Laso treated mice compared with Z only treated mice. (**B** Body weight was not significantly different between groups. (**C-E**) Histologic feature of intervertebral disc in Z (**c**), Z + E2 (**D**), and Z + Laso treated mice (**E**). **F** Histologic severity score of intervertebral disc. (**G-I**) Histologic features of ankle joint of Z (**G**), Z + E2 (**H**), and Z + Laso treated mice (**i**). (**J**) Histologic severity score of ankle joint. Values are mean ± standard error of the mean (*n* = 16 mice per group, except histologic severity score of intervertebral disc. Among 16 mice tails, 4 were used as histologic analysis and 12 were used for local mRNA expression). Analyses were performed using GraphPad Prism (version 5.0; GraphPad Software Inc., La Jolla, CA, USA, https://graphpad-prism.software.informer.com/5.0/). *p < 0.05, **p < 0.01, ***p < 0.001, ****p < 0.0001.

Arthritis development was assessed weekly during the experiment. After the zymosan injection, arthritis was developed and clinical arthritis scores had increased up to maximal score in zymosan group. A comparison of the arthritis severity over time revealed that E2 and Laso inhibited the arthritis development in SKG mice. Clinical score was significantly lower in the zymosan + E2-treated and zymosan + Laso treated mice than the zymosan treated group (Fig. 1A). Body weight was not significantly different between groups (Fig. 1B).

Histologic analysis was performed at 6 weeks after zymosan injection. Inflammation of the intervertebral discs of the tail and ankle joints was observed in zymosan-treated SKG mice (Fig. 1C,G). The degree of inflammation of the tail and ankle in the zymosan + E2 treated mice (Fig. 1D,H) was milder than zymosan-treated mice. The tail and ankle of the zymosan + Laso-treated mice (Fig. 1E,I) had few inflammatory cell infiltrations compared to the zymosan-treated mice. Histologic scores for intervertebral disc (Fig. 1F) and ankle joint (Fig. 1J) were significantly lower in zymosan + E2 and zymosan + Laso treated mice than zymosan treated mice. There was no significant difference between the zymosan + E2 treated mice and zymosan + Laso treated mice.

### Laso treatment reduced ^18^F-FDG uptake in the micro-PET/CT imaging

**Figure 2:**
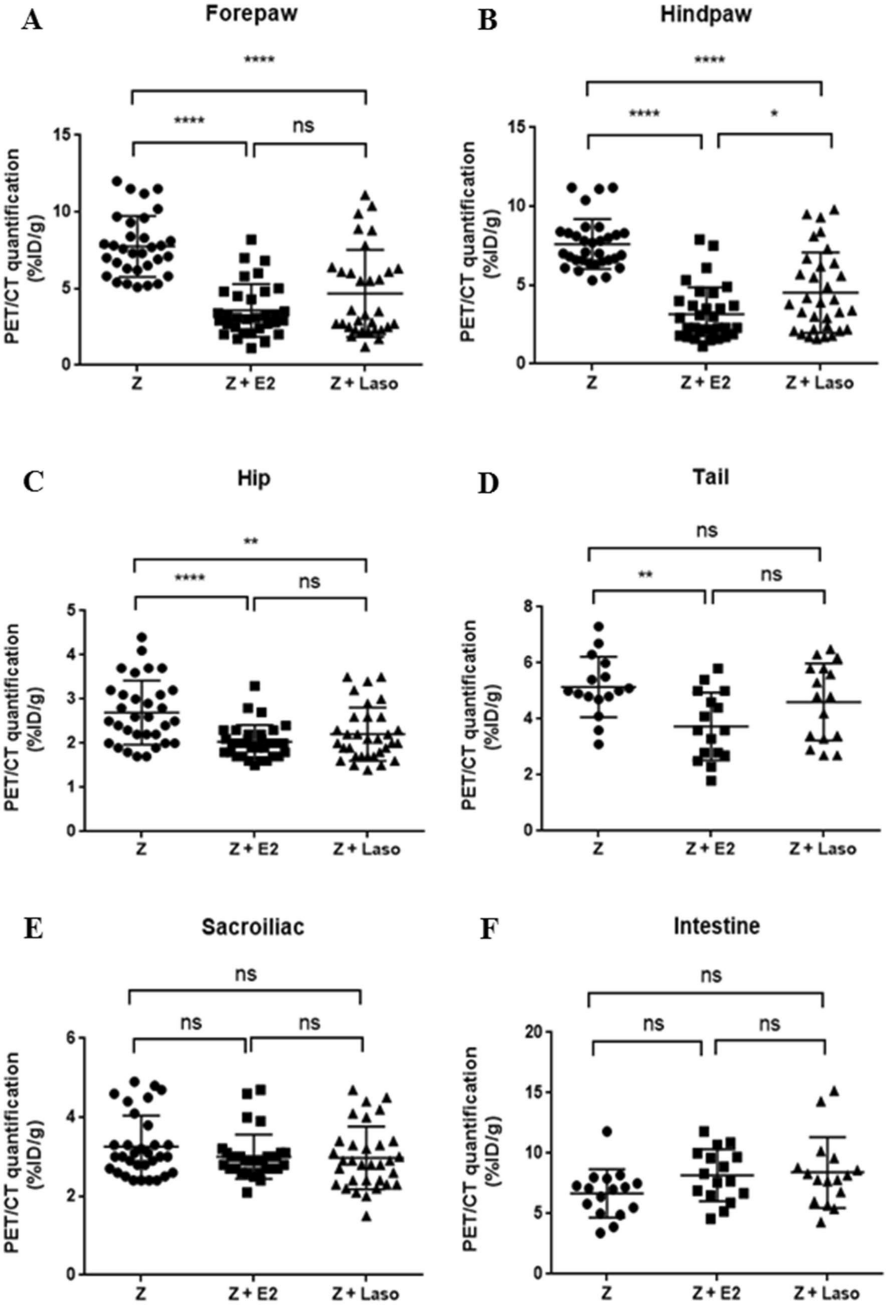
Measurement of ^18^F-fluorodeoxyglucose uptake. **A** Quantification of regions of interest in forepaw of 6 weeks after zymosan (Z) injection, Z + 17β-estradiol (E2) treated mice, and Z + lasofoxifene (Laso) treated mice. **B-F** Hindpaw (**B**), hip (**C**), tail (**D**), sacroiliac joint (**E**), and intestine (**F**). Values are mean ± standard error of the mean (*n* = 16 mice per group). Analyses were performed using GraphPad Prism (version 5.0; GraphPad Software Inc., La Jolla, CA, USA, https://graphpad-prism.software.informer.com/5.0/). *p < 0.05, **p < 0.01, ***p < 0.001, ****p < 0.0001. *PET/CT* positron emission tomography/computed tomography.

E2 and Laso treatment significantly reduced ^18^F-FDG uptake. The amount of ^18^F-FDG uptake of the forepaw (Fig. 2A), hindpaw (Fig. 2B), and hip joints (Fig. 2C) was significantly lower in the both zymosan + E2 treated mice and zymosan + Laso treated mice compared to the zymosan treated mice. ^18^F-FDG uptake of tail was significantly lower in the zymosan + E2 treated mice compared to the zymosan treated mice. But there was no significant difference between zymosan + Laso treated mice and zymosan treated mice (Fig. 2D). In the sacroiliac joint and intestine, there was no significant difference in the amount of ^18^F-FDG uptake between three groups (Fig. 2E,F).

### Expression of IL-17A, TNF-α, and IFN-γ was significantly reduced in zymosan + Laso treated mice

**Figure 3:**
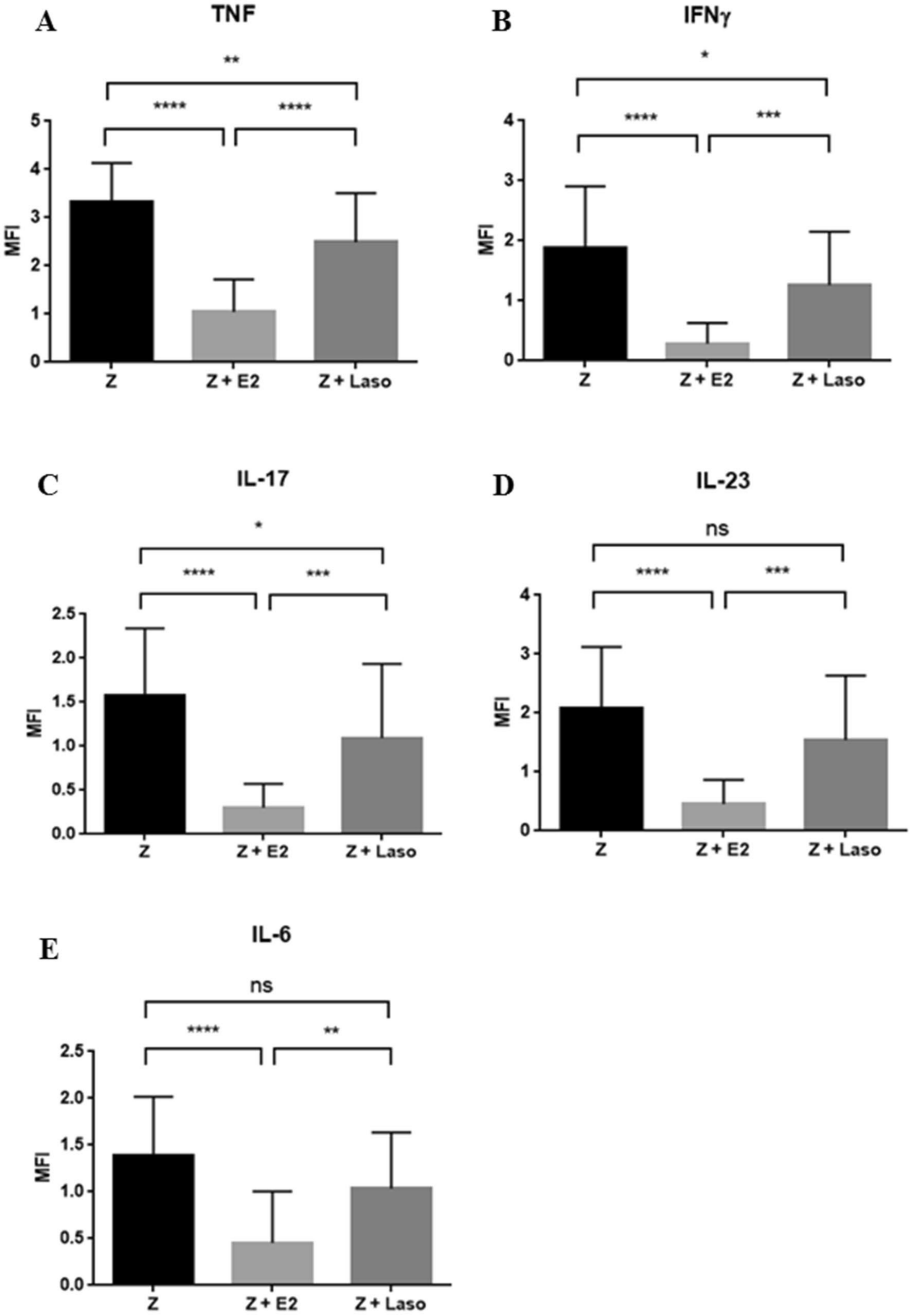
Local mRNA expression in hindpaw, forepaw, and tail was analyzed. Gene expression was calculated by median fluorescence intensity (MFI). **A-E** TNF-α (**A**), interferon gamma (IFN-γ) (**B**), IL-17 (**C**), IL-23 (**D**), IL-6 (**E**). Values are mean ± standard error of the mean (*n* = 16 mice per group). Analyses were performed using GraphPad Prism (version 5.0; GraphPad Software Inc., La Jolla, CA, USA, https://graphpad-prism.software.informer.com/5.0/). *p < 0.05, **p < 0.01, ***p < 0.001, ****p < 0.0001. *Z* zymosan, *E2* 17β-estradiol, *Laso* lasofoxifene.

Local gene expression in the tissue of hindpaws and forepaws were analyzed at 6 weeks after zymosan injection. Expression of TNF-α, IFN-γ, and IL-17A was significantly reduced in both the zymosan + E2 and zymosan + Laso mice compared with the zymosan treated mice (Fig. 3A-C). Expression of IL-23 and IL-6 was significantly reduced in zymosan + E2 treated group compared with the zymosan treated mice (Fig. 3D,E). There was no statistical significance of the expression of IL-23 and IL-6 between zymosan + Laso and zymosan treated mice. Zymosan + E2 treatment group showed decreased expression of all cytokines than in the zymosan + Laso treated group.

### BMD was increased in the zymosan + Laso treated mice

**Figure 4:**
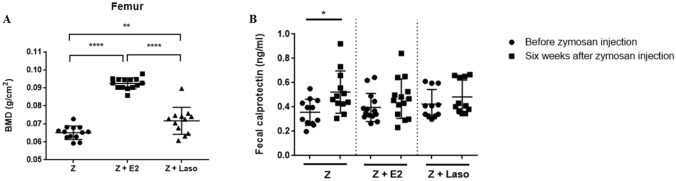
Bone mineral density (BMD) and fecal calprotectin levels between groups. (**A**) BMD was analyzed in femur. (**B**) Calprotectin levels at before zymosan (Z) infection and 6 weeks after Z injection in each group (*n* = 12–14 mice per group). Analyses were performed using GraphPad Prism (version 5.0; GraphPad Software Inc., La Jolla, CA, USA, https://graphpad-prism.software.informer.com/5.0/). *p < 0.05, **p < 0.01, ***p < 0.001, ****p < 0.0001. *E2* 17β-estradiol, *Laso* lasofoxifene.

BMD was detected by DXA. Femur were assessed at 6 weeks after zymosan injection (Fig. 4A). BMD was significantly increased in both zymosan + E2 and zymosan + Laso treated mice compared with the zymosan group. BMD was highest in zymosan + E2 treated mice among groups.

### Lasofoxifene treatment suppressed calprotectin in the fecal sample

Fecal calprotectin levels were assessed between before zymosan injection and 6 weeks after zymosan injection (Fig. 4B). Fecal calprotectin was significantly increased in zymosan-treated group at 6 weeks after zymosan injection compared with before zymosan injection. There were no significant changes in the fecal calprotectin levels in zymosan + E2 and zymosan + Laso treated mice.

### Altered microbiota diversity with the zymosan, E2, and Laso treatment

**Figure 5:**
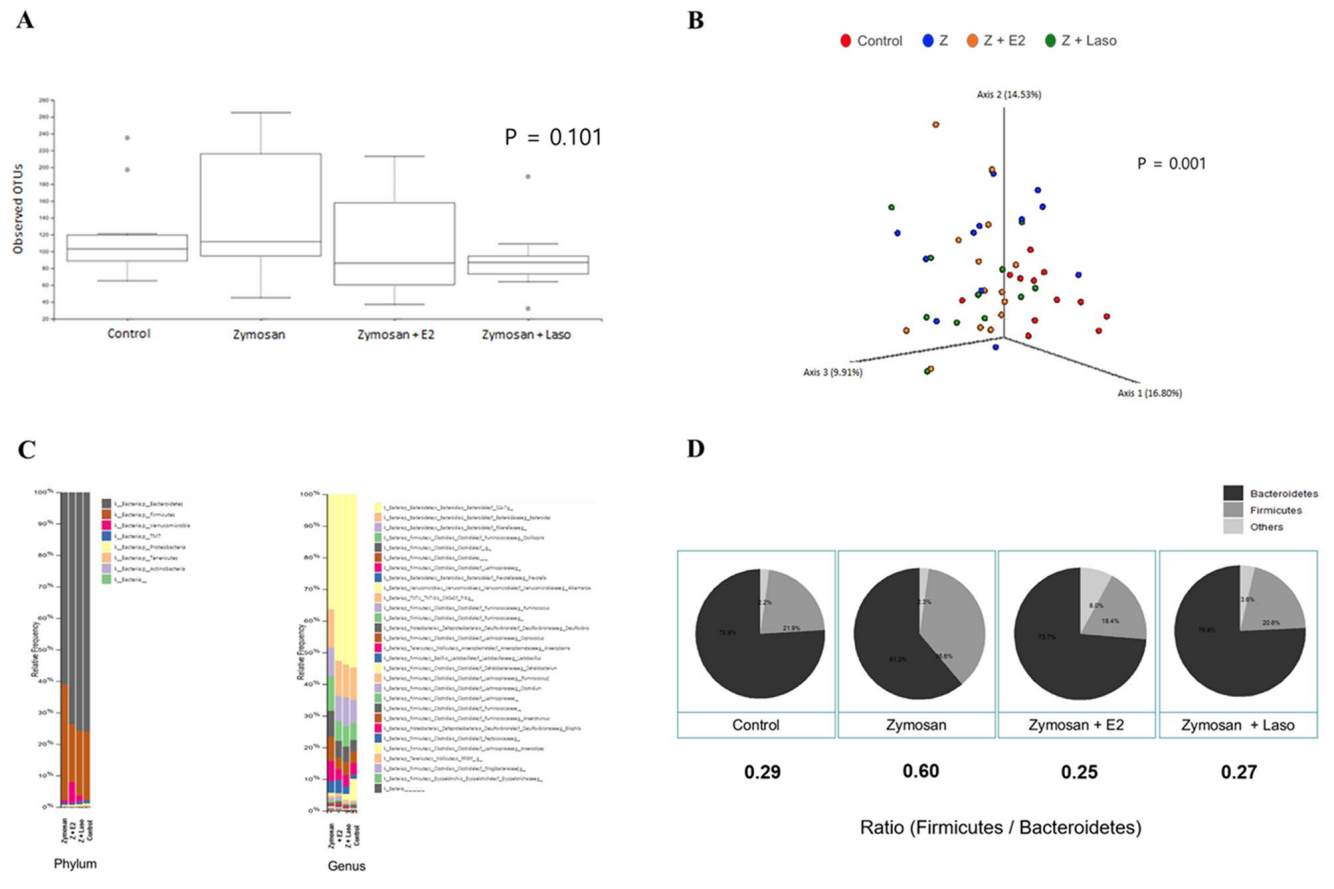
Bacterial composition of the gut microbiota. (**A**) α-diversity of gut microbiota between groups are measured by Shannon diversity index. The horizontal line inside the box represents the median, and the bottoms and tops of the boxes represent the 25th and 75th percentiles, respectively. Whiskers represent the lowest and highest values within the 1.5 interquartile range, respectively. Outliers are shown as dots. There was no statistical significance by Kruskal–Wallis test. (**B**) β-diversity, represented by principal coordinate analysis, was determined by Bray–Curtis distance matrices. Statistical significance was calculated using permutation multivariate analysis of variance test for β-diversity. (**C**) Global distribution of gut microbiota at phylum and genus levels. Subgroups are labelled on the X-axis and expressed as the relative operational taxonomic unit (OTU) abundance for each group. (**D**) *Firmicutes/Bacteroidetes* (F/B) ratio in phyla level were determined in control, zymosan (Z), Z + 17β-estradiol (E2), and Z + lasofoxifene (Laso) treated group (*p* = 0.059). F/B ratio was of Z group was lower than other three groups (*p* = 0.073). Analysis were performed using QIIME2 software (https://qiime2.org) and R studio (https://www.rstudio.com).

Fecal microbiota was analyzed. Control group was defined as fecal samples before the zymosan injection, which means normal healthy control (*n* = 12). Zymosan group refers to stool samples collected just before sacrifice, 6 weeks after zymosan administration (*n* = 12). Zymosan + E2 group refers to stool samples collected from the zymosan + E2 treated group at the time of sacrifice (*n* = 14). Zymosan + Laso group refers to stool samples collected from the zymosan + Laso treated group at the time of sacrifice (*n* = 12). We analyzed the bacterial fraction of microbiota using 16S sequencing. The α-diversity assessed by the number of observed OTUs was not significantly different between three groups (Fig. 5A). Beta diversity, assessed by bray-cutis was significantly different between the groups (Fig. 5B). Beta diversity of the gut microbiota was significantly different for control versus zymosan treated group, control versus zymosan + E2 treated group, control versus zymosan + Laso treated group, zymosan versus zymosan + E2 treated group, and zymosan versus zymosan + Laso treated group. However, there was no significant difference between zymosan + E2 and zymosan + Laso treated groups (Additional file 1).

### Difference of firmicutes/bacteroidetes ratio between groups

Figure 5C shows the global composition of bacterial microbiota at the phylum and genus level. The microbiota was dominated by bacteria from *Bacteroidetes* and *Firmicutes*. In phyla level, an abundance of *Firmicutes* was increased in the zymosan group compared with other groups. *Bacteroidetes/Firmicutes* (F/B) ratio of zymosan + E2 or zymosan + Laso treated group was similar to the healthy control group (Fig. 5D). Although there was no significance of B/F ratio between groups (*p* = 0.059), F/B ratio was higher in zymosan treated group compared with the other three groups (control, zymosan + E2, zymosan + Laso treated group).

### Specific bacteria were differentially enriched between groups

**Figure 6:**
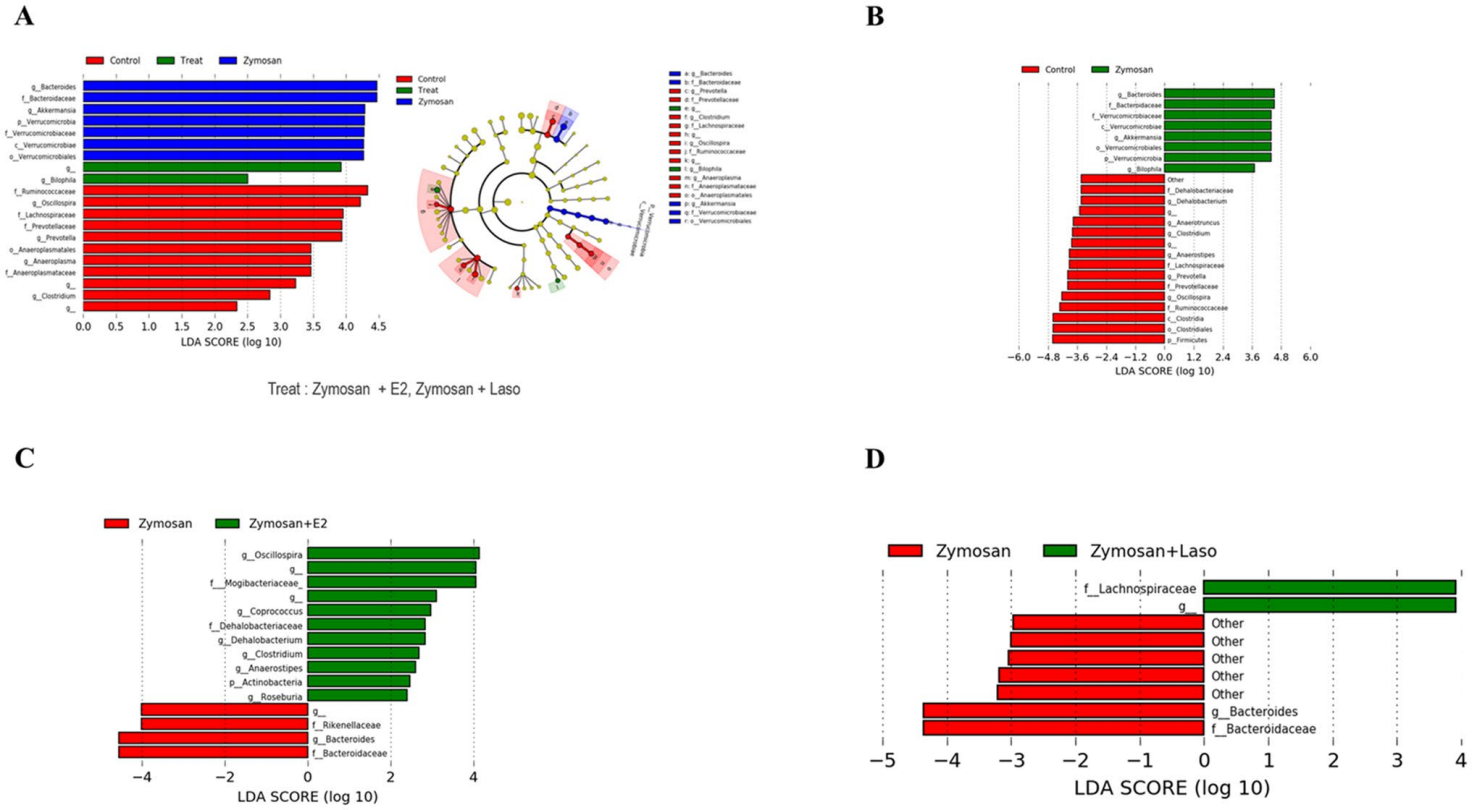
Linear discriminant analysis (LDA) effect size analysis identified the taxa with the greatest differences in abundance between groups. (**A**) LDA effect size analysis of subjects with control, treat, and zymosan (Z) treated group. Treat group refers to both Z + 17β-estradiol (E2) and Z + lasofoxifene (Laso) treated group. Cladogram shows the relationships among differentiating taxa between groups. (**B**) Taxa enriched in control group are indicated by a negative LDA score (red), and Z group enriched taxa are indicated by a positive score (green). (**C**) Taxa enriched in Z group are indicated by a negative LDA score (red), and taxa enriched in Z + E2 treated group are indicated by a positive LDA score (green). (**D**) Taxa enriched in Z group are indicated by a negative LDA score (red), and taxa enriched in Z + Laso treated group are indicated by a positive LDA score (green). The significant log LDA score > 2.0 for all taxa. Analysis were performed using QIIME2 software (https://qiime2.org) and LEfSe (https://twbattaglia.gitbooks.io/introduction-to-qiime/content/lefse.html). *g* genus, *f* family, *o* order.

Linear discriminant analysis using LEfSe were performed to determine whether specific individual bacterial taxa were differentially enriched between groups. Zymosan was characterized by an increase in *Bacteroides, Akkermansia,* and *Verrucomicrobia*. Zymosan + E2 or zymosan + Laso treated group were characterized by a greater abundance of *Bilophila*. *Ruminococcaceae, Oscillospira, Prevotella, Anaeroplasmata,* and *Clostridium* were enriched in control group (Fig. 6A). When compared between control group and zymosan treated group, zymosan treated group had a higher abundance of *Bacteroides, Verrucomicrobiaceae, Akkermansia,* and *Bilophila* (Fig. 6B). Control group had an increase in *Dehalobacteriaceae, Anaerotruncus, Clostridium, Anaerostipes, Lachnospiraceae, Prevotella, Oscillospira, Ruminococcaceae, Clostridia,* and *Firmicutes. Oscillospira, Mogibacteriaceae, Coprococcus, Dehalobacterium, Clostridium, Anaerostipes, Actinobacteria,* and *Roseburia* were enriched in zymosan + E2 treated group, while *Rikenellaceae*, and *Bacteroides* were enriched in zymosan group (Fig. 6C). *Lachnospiraceae* was enriched in zymosan + Laso treated group (Fig. 6D). When compared zymosan versus zymosan + E2 or zymosan + Laso treated group in genus level, relative abundance of *Oscillospira* and *Clostridium* was significantly higher in E2 or Laso treated group compared with zymosan group (Additional file 2).

## Discussion

We investigated the effect of SERM Laso on the disease activity of SpA. We previously reported that estrogens attenuate disease activity of SpA using an animal model^7^. In this study, we demonstrated that SERM, like estrogens, suppressed joint inflammation and increased BMD. The results of the present study correspond well with those of the earlier study which reported that SERM, both Laso and bazedoxifene, inhibit joint inflammation and osteoporosis in ovariectomized collagen-induced arthritis (CIA) animal model^14^. SERMs are known to stimulate estrogenic actions in tissues such as the liver, bone and cardiovascular system but known to block estrogens action where stimulation is not desirable, such as in the breast and the uterus. SERMs are approved for the treatment of postmenopausal osteoporosis. It has been suggested that inflammation plays a role in postmenopausal osteoporosis. Estrogens withdrawal is associated with increased production of proinflammatory cytokines, including IL-1, TNF-α, and IL-6, and these proinflammatory cytokines associated with increased osteoclastic bone resorption^15^. T cells are involved in the pathology of postmenopausal osteoporosis. Estrogens deficiency induced bone loss by increasing T cell production of TNF-α^16^. SERM might have anti-inflammatory effect on other diseases besides osteoporosis. SERM have anti-proliferative, anti-inflammatory effect in benign prostate hyperplasia in vitro studies and in vivo animal studies^17^. Suuronen et al.^18^ reported that SERM, tamoxifen, raloxifene, induced the anti-inflammatory response in acute mouse model and rat microglial cells though SERM induced modulation of lipopolysaccharide-activated pro-inflammatory signaling cascades. In postmenopausal women with osteoporosis, lasofoxifene 0.5 mg/day treatment showed a significant reduced risk of major coronary heart disease^19^. Low dose tamoxifen lower C-reactive protein levels in healthy women aged 35 to 70 years^20^.

In the present study, anti-inflammatory effect of Laso seems to less potent than E2. Although local gene expression of inflammatory cytokines including TNF-α, IFN-γ, IL-17, IL-23, and IL-6 in the joint was significantly lower in zymosan + Laso treated group than zymosan treated group, the amount of cytokine expression of zymosan + Laso treated group were higher than zymosan + E2 treated group. Bernardi et al.^21^ characterized immunomodulatory effect of SERM on T cell dependent inflammation compared with estrogens. E2 reduced thymus weight and decreased proportion of early T cell progenitor cells and suppressed T cell-dependent delayed-type hypersensitivity reaction. However, Laso decreased thymus weight but no effect on T cell population in the thymus or on inflammation in delayed-type hypersensitivity reaction. SERM might have less inflammatory effect compared with E2, other factors might affect on results of the current study. We used a commercially available estrogen pellet for E2 infusion. However, there was no commercial SERM pellet and we used 45 days releasing mini-osmotic pump. Even with the largest mini-osmotic pump, we were able to put as much as one-third of the planned SERM dose. Thus, SERM was used at a lower concentration compared with the E2. Furthermore, the pump is 3 cm long and hard. Some mice had a wound problem at the pump insertion site and some were infected. Pump deteriorated condition and caused soft tissue infection, which might have affected the results of the study.

Calprotectin is major antimicrobial leukocyte proteins. Inflammation cause neutrophil activation, which results in the release of calprotectin into body fluid including feces^22^. Fecal calprotectin is used as a biomarker for inflammatory bowel diseases such as Crohn`s disease or ulcerative colitis^23^. Fecal calprotectin levels showed a significant correlation with disease activity parameters of AS, including the Bath Ankylosing Spondylitis Disease Activity Index, Bath Ankylosing Spondylitis Functional Index, C-reactive protein and erythrocyte sedimentation rate^24^. In the current study, fecal calprotectin level was significantly increased after zymosan injection compared with before zymosan injection. On the other hand, there was no significant change in the zymosan + E2- or zymosan + Laso- treated group. The results of this study suggest that E2 or SERM suppressed inflammation in the intestine as well as joint.

*Firmicutes* and *Bacteroidetes* were the most common bacterial phyla in the human microbiota. In general, F/B ratio is regarded to be of significant relevance in the composition of human gut microbiota^25^. Although there was no statistical significance, we identified trends that relative abundance of phylum *Firmicutes* was increased and *Bacteroidetes* was decreased in zymosan treated mouse compared with control group. However, F/B ratio of zymosan + E2 or zymosan + Laso treated group was similar to the control group. Our study was consistent with other studies showed that estradiol supplementation showed significantly lower level of the F/B ratio compared with control group in a male mouse of colorectal cancer model^26^. E2 treatment increased the relative abundance of *Bacteroidetes* in leptin-deficient mice^27^. Rogier et al.^28^ reported that the intestinal microbiota of naïve mice was dominated by the phylum *Bacteroidetes*. However, in the immune-priming phase of CIA mouse, phylum *Firmicutes* dominated and abundance of *Bacteroidetes* was decreased, which suggests that intestinal microbiota alteration precede the onset of arthritis.

In current study, although α-diversity was not significantly different between group regardless of E2 treatment, β-diversity was significantly different between groups. E2 or Laso treatment altered β-diversity, but there was no significant difference between zymosan + E2 treated group and zymosan + Laso treated group. A previous study showed that E2 treatment was associated with lower species evenness and E2 treatment also effect on β-diversity in leptin-deficient mice^27^. In male mice of colorectal cancer model, α-diversity, observed OTU count, was increased in E2 supplemented male. And β-diversity was significantly distinguished based on E2 treatment^26^. Fecal microbial diversity was correlated with urinary estrogens in postmenopausal women^29^. Estrogen receptor (ER), including ER-α and ER-β, are present in the intestine and cells of the immune system. Estrogens treatment dramatically improved stool scores and histologic scores in HLA-B27 transgenic rat model of inflammatory bowel disease^30^. These results suggest that estrogens could cause changes in the gut microbiota.

A certain number of intestinal commensal bacteria including *Bacteroides, Clostridium, Bifidobacterium,* and *Lactobacillus* are effect on the host mucosal immune system^31^. We found that *Bacteroides, Akkermansia,* and *Verrucomicrobia* were increased in zymosan injected mice. Our study was consistent well with other recent studies showing that *Akkermansia, Bacteroides*, and *Verrucomicrobia* were significantly increased in proteoglycan induced AS mouse model^32^. We found that *Oscillospira* was significantly increased in E2 or Laso treated mice. *Oscillospira* was significantly higher in the gut microbiota of females than that of male subjects^33^. There were several studies of implicating gut microbiota in patients with AS. In patients with AS, *Lachnospiraceae, Ruminococcaceae, Rikenellaceae, Porphyromonadaceae, Bacteroidaceae* were increased in terminal ileum biopsy specimen^34^. *Dialister* in ileal biopsy tissue from patients with SpA was positively correlated with AS disease activity scores^35^. While many common genera are found in the human and mouse intestine, these differ strongly in abundance and in total only 4% of the bacterial genes are found to share identity^36^. Since human microbiota is very different from that of mice due to genetics and dietary pattern, there are limitations in interpreting the results of microbiota experiment using murine model.

The rearrangement of gut microbiota might have some influence on the disease expression of human disease^37^. Gut lining is very tight together and separate bacterial protein, toxin, and undigested food particle in the gut from the gut immune system. Imbalanced gut bacteria or bacterial toxin such as lipopolysaccharide damaged the gut lining and becomes leaky. These products get through the gut lining and stimulate gut immune system. Intestinal lymphocytes from inflamed gut bind well to the vessels in synovial membrane of the joint using homing receptors and their corresponding endothelial ligands^38^. Under inflammatory condition, non-gut specific adhesion ligands (CD44, VLA-4, FLA-1) of T cells from the gut-associated lymphoid organ are highly expressed, and these T cells move from gut to joint and binds to the adhesion ligands (VCAM-1, VAP-1, ICAM) of the synovial endothelial cells in SpA^39,40^. SKG mice harboring microbiota from rheumatoid arthritis patients showed an increased number of intestinal Th17 cells and developed severe arthritis when treated with zymosan compared with mice harboring with microbiota from healthy control^41^. It suggests that dysbiosis trigger arthritis via activation of autoreactive T cells in the intestine. Dysbiosis contributes to the development of gut inflammation. Manipulating of gut microbiome might prevent or treat leaky gut and arthritis development.

In the previous study about zymosan induced SKG mice model, we found that arthritis occurred earlier in the female mice than in the male mice^10^. Ultimately, almost all of the mice of both sexes developed arthritis 12 weeks after zymosan injection. Although SpA affects males in larger proportion than females, we used female mice for this experiment. Because experiment requires controlling the concentration of estrogen or lasofoxifene, it would be more appropriate to use female mice than male mice. Furthermore, SERM was approved for postmenopausal women only. There are limited numbers of studies investigating SERM treatment in males. Raloxifene treatment improved BMD and decreased fracture in male patients with gonadal suppression therapy due to prostate cancer^42^. Raloxifene treatment prevented bone loss in orchidectomized rats^43^. There has been no experiment for young male without gonadal suppression. Although the proportion of postmenopausal women in patient with SpA is small, SERM may be effective as a treatment for postmenopausal women with SpA.

In conclusion, we demonstrated that SERM Laso inhibited joint inflammation and enhanced BMD in SKG mice, a model of SpA. Laso also affected gut microbiota characterized by alterations in composition and biodiversity. We suggest the possibility that SERM treatment could change the gut microbiota, which results in reducing the risk of developing SpA. This study provides new knowledge regarding that selected SpA patients, especially postmenopausal women, could benefit from SERM treatment.

## Methods

### Experimental mice

SKG mice were purchased from CLEA Japan and were housed in a specific-pathogen-free facility under climate-controlled conditions. Mice were provided with water and standard diet ad libitum. All procedures were approved by the Institutional Animal Care and Use Committee at Samsung Medical Center, Sungkyunkwan University School of Medicine. All animal experiments were performed according to the guidelines issued by the Institutional Animal Care and Use Committee in accordance with the National Institute of Health (NIH) guidelines. This study was carried out in compliance with the ARRIVE guidelines^44^.

### Lasofoxifene or 17β-estradiol (E2) treatment

Experiments were performed in three groups: zymosan treated group, zymosan + 17β-E2 treated group, and zymosan + Laso treated group. Eight-week-old female SKG mice underwent an ovariectomy under anesthesia 2 weeks before arthritis induction. One week later after ovariectomy, slow-releasing pellets of E2 (0.72 mg, 60 days release; Innovative Research, Sarasota, FL, USA) were implanted subcutaneously on the neck scruff of mice in the E2-treated group^7,45^. For continuous Laso delivery, subcutaneously implanted ALZET osmotic pump (Alza Corp., Palo Alto, CA, USA) technique was used. One week later after ovariectomy, mice underwent subcutaneous implantation of an ALZET osmotic pump that infused Laso 1.5 µg/day for 6 weeks. Laso tartrate was mixed with DMSO (solubility 10 mg/ml). The nominal reservoir volume of ALZET osmotic pump (Model 2006; 6 weeks duration) was 200 uL and the release rate was 0.15 ul/h.

### Induction of arthritis and scoring of clinical arthritis

Arthritis was induced 2 weeks after ovariectomy or 1 week after E2 pellet or Laso osmotic pump implantation. Zymosan A (Sigma-Aldrich, St Louis, MO, USA) was suspended in PBS and incubated for 10 min in boiling water. Then the zymosan A solution was injected intraperitoneally into 10-week-old mice in each of the three groups (3 mg/mouse): zymosan, zymosan + E2, zymosan + Laso. ^18^F-fluorodeoxyglucose (^18^F-FDG) small-animal positron emission tomography (PET)/computed tomography (CT) and cytokine analysis were performed. Clinical scores were monitored following a previously published system^8^: 0, no swelling or redness; 0.1, swelling or redness of the digits; 0.5, mild swelling and/or redness of the wrists or ankle joints; and 1, severe swelling of the larger joints. Scores for the affected joints were totaled for each mouse. The maximum possible score was 6. Clinical scores were monitored weekly, and mice were sacrificed 6 weeks after zymosan injection.

### ^*18*^*F-FDG PET/CT acquisition and image analysis*

Mice were fasted for 5 h before micro-PET/CT studies. A 1.8-MBq injection of ^18^F-FDG mixed with isotonic saline in a total volume of 150 μL was administered through a tail vein. Imaging was performed 1 h later under isoflurane anesthesia without respiratory gating. Micro-PET images of mice were acquired using an Inveon micro-PET/CT scanner (Siemens Medical Solutions, Malvern, PA, USA) at the Center for Molecular and Cellular Imaging, Samsung Biomedical Research Institute (Seoul, Korea)^7^. Acquisition of non-enhanced CT images was followed by PET imaging. Micro-PET images obtained were reconstructed using three-dimensional (3D)-ordered subset expectation maximization and then processed using Siemens Inveon Research Workplace 4.1. The 3D regions of interest (ROIs) were drawn over the joints using a threshold range of 70% to 100% of the maximum intensity, and the average signal level in the ROIs was measured^7^. In the case of the intestine, the ROIs were measured in the abdomen excluding the kidneys and bladder, and percentage injected dose per gram (%ID/g) was measured at a threshold value of 70% to 100%^7^. Image counts/pixel/s were converted to radioactivity concentrations that were then corrected for injected radioactivity. The ROIs were measured by a technician who blinded to all clinical data. Signal levels are expressed as the mean ± standard deviation %ID/g.

### Histopathological examination

Mice were anesthetized and euthanized after 6 weeks of zymosan injection. Mouse joint tissues were fixed in 10% formalin, decalcified in ethylenediaminetetraacetic acid, and embedded in paraffin. Sections were deparaffinized using xylene, dehydrated in a graded series of alcohol solutions, and stained with hematoxylin and eosin. Histologic features of the mouse joints were scored as described previously^46^: 1–4, where 1 = few infiltrating immune cells, 2 = 1–2 small patches of inflammation, 3 = inflammation throughout the joint, and 4 = inflammation in soft tissue/entheses/fasciitis. Histologic features of the tail were scored on a scale of 1–4, where 1 = few infiltrating immune cells, 2 = mild inflammation of the discs or along the vertebrae (0–30% of discs), 3 = inflammation of the discs and/or along the vertebrae (30–70% of discs), and 4 = inflammation in > 70% of the discs and along the vertebrae.

### RNA isolation and QuantiGene 2.0 Plex assay

Total RNA was extracted from the hind paws, forepaws, and tail using a total RNA kit (QuantiGene sample processing kit) according to the manufacturer’s protocol. Target hybridization and signal amplification were performed according to the manufacturer’s protocol for fresh tissues (QuantiGene 2.0 Plex assay). Signal was detected using a Luminex 100/200 system and reported as median fluorescence intensity (MFI), which is proportional to the number of target RNA molecules present in the sample^7^. Gene expression was first calculated by determining the average signal (MFI) for all genes, and average background signals were subtracted for each gene. Levels of gene expression were then determined by dividing each test gene signal (background subtracted) by the normalization gene signal (background subtracted). Each sample was assayed using technical duplicates. Target genes were tumor necrosis factor (TNF)-α, IL-6, interferon (IFN)-γ, IL-17A, IL-23, and IL-6.

### Dual-energy X-ray absorptiometry (DXA) test

DXA testing was conducted to determine the bone mineral density (BMD) of the mouse. The DXA images were acquired using a PIXImus 2 mouse densitometer (GE Lunar PIXImus; GE Lunar, Madison, WI, USA) after sedation. The mice were anesthetized as in the ^18^F-FDG PET/CT test, positioned for the dorsal–ventral view, including the tail on a DXA tray, and irradiated by X-ray with lower energy (35 kV) and with high energy (70 kV). The DXA equipment was calibrated with a plastic phantom mouse provided by the manufacturer at the 1320 threshold. BMD were calculated in the femur area by the equipment computer counting equation. BMD values are obtained automatically from the DEXA scan in the femur. Bone area measurement is generated by outlining or specifying the limits or dimensions of the entire skeletal bone regions of the body, excluding the head, as regions of interest (ROI) following a full body X-ray scan. Bone mineral content (BMC) is generated from PIXImus density scans which are assessed for accuracy using a set of 0.0 mg to 2,000 mg of hydroxyapatite standards. The BMD were calculated by dividing the BMC by the bone area.

### Fecal sample collection and DNA isolation

Fresh stool samples were collected twice, before zymosan injection and 6 weeks after zymosan injection. Fresh stool sample were stored frozen immediately and kept at -80℃ before being processed. Fecal DNA was collected using the QIAamp fast DNA stool mini kit (QIAGEN, Hilden, Germany) following manufacturer’s instructions.

### 16S ribosomal RNA (16S rRNA) gene sequencing

The V3–V4 region of the 16S rRNA gene was amplified using the 341F and 805R primers with added Illumina adaptor overhang sequences, 341F (5' TCG TCG GCA GCG TCA GAT GTG TAT AAG AGA CAG CCT ACG GGN GGC WGC AG 3') and 805R (5' GTC TCG TGG GCT CGG AGA TGT GTA TAA GAG ACA GGA CTA CHV GGG TAT CTA ATC C 3')^47^. Amplicons were purified with a magnetic bead-based clean-up system (Agencourt AMPure XP; Beckman Coulter, Brea, CA, USA). Indexed libraries were prepared by limited-cycle polymerase chain reaction using Nextera technology, further cleaned up, and pooled at equimolar concentrations. The final library was denatured with 0.2 N NaOH and diluted to 6 pM with a 20% PhiX control. Sequencing was performed on Illumina MiSeq platform (Illumina, San Diego, CA, USA) using a 2 × 300 bp paired-end protocol, according to the manufacturer’s instructions.

### 16S rRNA gene sequence analysis

The FastQ files of the paired-sequenced data were denoised and operational taxonomic unit (OTU) picking was performed in paired method of quality filtered in DADA2 plug-in within the QIIME2 pipeline (https://qiime2.org)^48^. QIIME2 is python based software that we used to determine metagenome data OTU, taxonomy, and diversity with sequencing data from each sample. All sequence samples were compiled and classified for each OTU and aligned them to reference counting taxonomy. Firstly compiled sequence data were grouped with those up to 97% similarity or 100 bp sequence likeness as representative sequence using cd-hit method to process with better quality samples. Then fast aligning best-matching sequences called Nast algorithm was used to map each OUT’s representative sequences to the reference and generate biome file nominating taxonomy lists. Biom file is an important intermediate result file to depict α, β-diversity and determine amount of each samples in certain OTU groups. Phylogeny tree file is another necessary data that create a phylogenetic tree from OTU sequences with fast tree algorithm and elaborate taxonomic analysis contingent upon score or adenoma stage. These two files are needed to exceed the core diversity process and error-prone if they do not match. Furthermore, count of read depth from sample data can be filtered before processing core diversity; however, we did not cut off any read-depth in case of scantiness of sample. Taxonomy lists are present at each level after core diversity, as well as taxonomic differences overscore, age and any of comparisons and α- and β-diversity analysis are also available to validate results. Linear discriminant analysis (LDA) effect size (LEfSe) method to support high-dimensional class comparisons with a focus on metagenomic analyses. LEfSe uses the significance of differences in OTUs in 2 groups that Bacterial taxa abundances.

### Statistical analysis

Two-way analysis of the variance (ANOVA) was used to analyze treatment effects on clinical scores over time. Tukey’s post-hoc test was performed to compare multiple means. The differences in α- and β-diversity among or between groups could be statistically evaluated using the Mann–Whitney U test, ANOVA. Mann–Whitney *U* test is a nonparametric test that allows two groups or conditions to be compared without assumption that values are normally distributed, which is used and more appropriate because the OTU distributions in microbiome data usually deviate from normality. ANOVA is used to test for significant differences between the means of more than two clinical groups. Statistical significance was indicated in false discovery rate set at 10%, and differences with a *p* value less than 0.05. Results are presented as mean ± standard error of the mean (SEM). All analyses were performed using SPSS software, version 19.0 (SPSS Inc., Chicago, IL, USA) and GraphPad Prism (version 5.0; GraphPad Software Inc., La Jolla, CA, USA). Statistical significance was indicated on figures as follows: *p < 0.05, **p < 0.01, ***p < 0.001, ****p < 0.0001.

## Supplementary Information

Supplementary Information 1.

Supplementary Information 2.

Supplementary Information 3.

## References

[1] Lee W (2007). Are there gender differences in severity of ankylosing spondylitis? Results from the PSOAS cohort. Ann. Rheum. Dis..

[2] Ortega Castro R (2013). Different clinical expression of patients with ankylosing spondylitis according to gender in relation to time since onset of disease. Data from REGISPONSER. Reumatol. Clin..

[3] Jeong H (2015). Clinical characteristics of nonradiographic axial spondyloarthritis in Korea: A comparison with ankylosing spondylitis. Int. J. Rheum. Dis..

[4] Taneja V (2018). Sex Hormones Determine Immune Response. Front. Immunol..

[5] Jimenez-Balderas FJ, Tapia-Serrano R, Madero-Cervera JI, Murrieta S, Mintz G (1990). Ovarian function studies in active ankylosing spondylitis in women. Clinical response to estrogen therapy. J. Rheumatol..

[6] Jeong H, Bea EK, Lee J, Koh EM, Cha HS (2015). Body mass index and estrogen predict radiographic progression in the spine in ankylosing spondylitis. Joint Bone Spine.

[7] Jeong H (2017). Estrogen attenuates the spondyloarthritis manifestations of the SKG arthritis model. Arthritis Res. Ther..

[8] Sakaguchi N (2003). Altered thymic T-cell selection due to a mutation of the ZAP-70 gene causes autoimmune arthritis in mice. Nature.

[9] Ruutu M (2012). Beta-glucan triggers spondylarthritis and Crohn's disease-like ileitis in SKG mice. Arthritis Rheum.

[10] Jeong H (2018). Spondyloarthritis features in zymosan-induced SKG mice. Joint Bone Spine.

[11] Sherlock JP (2012). IL-23 induces spondyloarthropathy by acting on ROR-γt+ CD3+CD4-CD8- entheseal resident T cells. Nat. Med..

[12] Breban M (2017). Faecal microbiota study reveals specific dysbiosis in spondyloarthritis. Ann. Rheum. Dis..

[13] Nakamura YK (2016). Gut microbial alterations associated with protection from autoimmune uveitis. Invest. Ophthalmol. Vis. Sci..

[14] Andersson A (2016). Selective oestrogen receptor modulators lasofoxifene and bazedoxifene inhibit joint inflammation and osteoporosis in ovariectomised mice with collagen-induced arthritis. Rheumatology (Oxford).

[15] Mundy GR (2007). Osteoporosis and inflammation. Nutr. Rev..

[16] Cenci S (2000). Estrogen deficiency induces bone loss by enhancing T-cell production of TNF-alpha. J. Clin. Invest..

[17] Garg M (2013). Selective estrogen receptor modulators for BPH: New factors on the ground. Prostate Cancer Prostatic Dis..

[18] Suuronen T (2005). Anti-inflammatory effect of selective estrogen receptor modulators (SERMs) in microglial cells. Inflamm. Res..

[19] Ensrud K (2010). Lasofoxifene and cardiovascular events in postmenopausal women with osteoporosis: Five-year results from the postmenopausal evaluation and risk reduction with lasofoxifene (PEARL) trial. Circulation.

[20] Bonanni B (2003). Effect of tamoxifen at low doses on ultrasensitive C-reactive protein in healthy women. J. Thromb. Haemost..

[21] Bernardi AI (2015). Selective estrogen receptor modulators in T cell development and T cell dependent inflammation. Immunobiology.

[22] Aomatsu T (2011). Fecal calprotectin is a useful marker for disease activity in pediatric patients with inflammatory bowel disease. Dig. Dis. Sci..

[23] Dhaliwal A (2015). Utility of faecal calprotectin in inflammatory bowel disease (IBD): What cut-offs should we apply?. Frontline Gastroenterol..

[24] Duran A (2016). Fecal calprotectin is associated with disease activity in patients with ankylosing spondylitis. Bosn. J. Basic Med. Sci..

[25] Ley RE, Turnbaugh PJ, Klein S, Gordon JI (2006). Microbial ecology: Human gut microbes associated with obesity. Nature.

[26] Song CH (2020). 17β-Estradiol supplementation changes gut microbiota diversity in intact and colorectal cancer-induced ICR male mice. Sci. Rep..

[27] Acharya KD, Gao X, Bless EP, Chen J, Tetel MJ (2019). Estradiol and high fat diet associate with changes in gut microbiota in female ob/ob mice. Sci. Rep..

[28] Rogier R (2017). Alteration of the intestinal microbiome characterizes preclinical inflammatory arthritis in mice and its modulation attenuates established arthritis. Sci. Rep..

[29] Fuhrman BJ (2014). Associations of the fecal microbiome with urinary estrogens and estrogen metabolites in postmenopausal women. J. Clin. Endocrinol. Metab..

[30] Harnish DC (2004). Beneficial effects of estrogen treatment in the HLA-B27 transgenic rat model of inflammatory bowel disease. Am. J. Physiol. Gastrointest. Liver Physiol..

[31] Reading NC, Kasper DL (2011). The starting lineup: Key microbial players in intestinal immunity and homeostasis. Front. Microbiol..

[32] Liu G, Ma Y, Yang Q, Deng S (2020). Modulation of inflammatory response and gut microbiota in ankylosing spondylitis mouse model by bioactive peptide IQW. J. Appl. Microbiol..

[33] Yuan X, Chen R, Zhang Y, Lin X, Yang X (2020). Sexual dimorphism of gut microbiota at different pubertal status. Microb Cell Fact.

[34] Costello ME (2015). Brief report: Intestinal dysbiosis in ankylosing spondylitis. Arthritis Rheumatol.

[35] Tito RY (2017). Brief report: Dialister as a microbial marker of disease activity in spondyloarthritis. Arthritis Rheumatol.

[36] Hugenholtz F, de Vos WM (2018). Mouse models for human intestinal microbiota research: A critical evaluation. Cell. Mol. Life Sci..

[37] Mu Q, Kirby J, Reilly CM, Luo XM (2017). Leaky gut as a danger signal for autoimmune diseases. Front. Immunol..

[38] Salmi M, Jalkanen S (2001). Human leukocyte subpopulations from inflamed gut bind to joint vasculature using distinct sets of adhesion molecules. J. Immunol..

[39] Fantini MC, Pallone F, Monteleone G (2009). Common immunologic mechanisms in inflammatory bowel disease and spondylarthropathies. World J. Gastroenterol..

[40] Hindryckx P (2011). Subclinical gut inflammation in spondyloarthritis is associated with a pro-angiogenic intestinal mucosal phenotype. Ann. Rheum. Dis..

[41] Maeda Y (2016). Dysbiosis contributes to arthritis development via activation of autoreactive T cells in the intestine. Arthritis Rheumatol.

[42] Smith MR, Fallon MA, Lee H, Finkelstein JS (2004). Raloxifene to prevent gonadotropin-releasing hormone agonist-induced bone loss in men with prostate cancer: A randomized controlled trial. J. Clin. Endocrinol. Metab..

[43] Ke HZ (2000). Lasofoxifene (CP-336,156), a selective estrogen receptor modulator, prevents bone loss induced by aging and orchidectomy in the adult rat. Endocrinology.

[44] Percie du Sert N (2020). The ARRIVE guidelines 2.0: Updated guidelines for reporting animal research. J. Physiol..

[45] Ingberg E, Theodorsson A, Theodorsson E, Strom JO (2012). Methods for long-term 17β-estradiol administration to mice. Gen. Comp. Endocrinol..

[46] Ruutu M (2012). β-glucan triggers spondylarthritis and Crohn's disease-like ileitis in SKG mice. Arthritis Rheum..

[47] Klindworth A (2013). Evaluation of general 16S ribosomal RNA gene PCR primers for classical and next-generation sequencing-based diversity studies. Nucleic Acids Res..

[48] Bolyen E (2019). Reproducible, interactive, scalable and extensible microbiome data science using QIIME 2. Nat. Biotechnol..

