# Supplementary figures and images for: Selective estrogen receptor modulator lasofoxifene suppresses spondyloarthritis manifestation and affects characteristics of gut microbiota in zymosan-induced SKG mice
Source: Sci Rep. 2021 Jun 7;11:11923. doi: 10.1038/s41598-021-91320-1 (PMC8184804; doi:10.1038/s41598-021-91320-1)

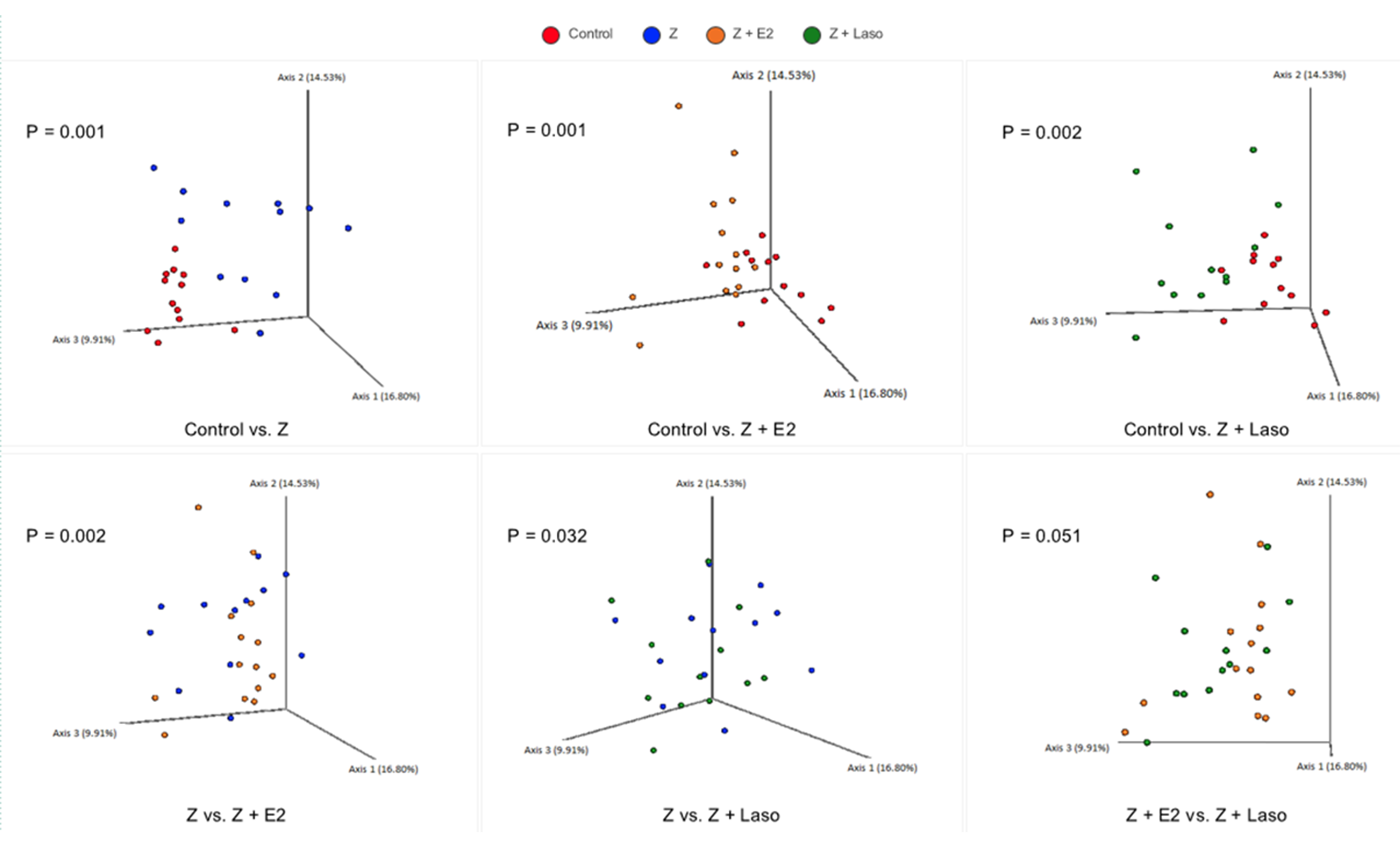

Supplement: Supplementary file 1 — Supplementary Information 1. [file 41598_2021_91320_MOESM1_ESM.tif]

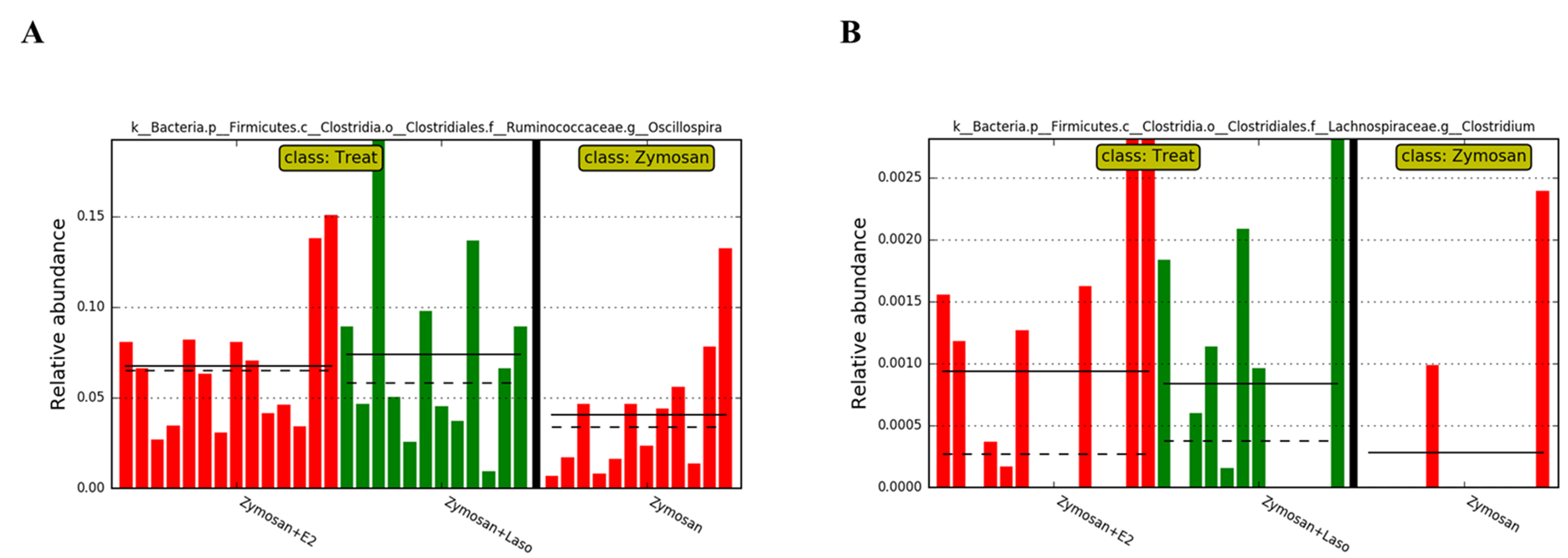

Supplement: Supplementary file 2 — Supplementary Information 2. [file 41598_2021_91320_MOESM2_ESM.tif]
