# Supplementary material for: Selective estrogen receptor modulator lasofoxifene suppresses spondyloarthritis manifestation and affects characteristics of gut microbiota in zymosan-induced SKG mice
Source: Sci Rep. 2021 Jun 7;11:11923. doi: 10.1038/s41598-021-91320-1 (PMC8184804; doi:10.1038/s41598-021-91320-1)
Supplement: Supplementary file 3 — Supplementary Information 3. [file 41598_2021_91320_MOESM3_ESM.docx]

**Supplementary information**

**File name:** Additional file 1

**File format:** DOC

**Figure S1.** β-diversity, represented by principal coordinate analysis, was determined by Bray-Curtis distance matrices. Permutation multivariate analysis of variance test for the dissimilarity of bacterial population structures was performed from samples of control vs. zymosan (Z) (*p* = 0.001), control vs. Z + 17β-estradiol (E2) (*p* = 0.001), control vs. Z + lasofoxifene (Laso) (*p* = 0.002), Z vs. Z + E2 (*p* = 0.002), and Z vs. Z + Laso (*p* = 0.032). There was no statistical significance between Z + E2 and Z + Laso treated group (*p* = 0.051)

**File name:** Additional file 2

**File format:** DOC

**Figure S2.** Relative abundance in zymosan (Z) vs. 17β-estradiol (E2) or lasofoxifene (Laso) treated group in genus level. Treat group defines both Z + E2 and Z + Laso treated group. Relative abundance of *Oscillospira* (**a**) and *Clostridium* (**b**) was significantly higher in treated group than Z treated group
